# Taijin Kyofusho and Social Anxiety and Their Clinical Relevance in Indonesia and Switzerland

**DOI:** 10.3389/fpsyg.2013.00003

**Published:** 2013-02-04

**Authors:** N. Vriends, M. C. Pfaltz, P. Novianti, J. Hadiyono

**Affiliations:** ^1^Department of Psychology, Division of Clinical Psychology and Psychotherapy, University BaselBasel, Switzerland; ^2^Faculty of Psychology, Gadjah Mada UniversityYogyakarta, Indonesia

**Keywords:** social anxiety, taijin kyofusho, cultural clinical psychology, self-construal, social phobia, treatment wish

## Abstract

**Background:** Taijin Kyofusho Scale (TKS) is an interpersonal fear to offend others and is defined by Diagnostic and Statistical Manual of Mental Disorders (DSM-IV) as a culturally bound syndrome that occurs in Japan and Korea. Recently, cases with TKS have also been recognized in other cultures. The present questionnaire study investigated self-report TKS symptoms and social anxiety symptoms, and their clinical relevance in an Indonesian and Swiss sample. It also investigated whether self-construal is associated with TKS and social anxiety, and if self-construal is a mediator of the expected association between cultural background and social anxiety and TKS symptoms.

**Method:** 311 Indonesian and 349 Swiss university students filled out the Liebowitz Social Anxiety Scale, the Taijin Kyofusho Scale, the Self-Construal Scale, self-report social phobia DSM-IV criteria, and rated their wish for professional help to deal with social fears.

**Results:** TKS and social anxiety symptoms were higher in the Indonesian than the Swiss sample. TKS symptoms were associated with clinical relevance in Indonesia, whereas in Switzerland only social anxiety symptoms were associated with clinical relevance. Independent self-construal was negatively associated and interdependent self-construal was positively associated with TKS and social anxiety symptoms. Interdependent self-construal mediated the association between cultural background and these symptoms.

**Discussion:** TKS might be a clinically relevant syndrome in all individuals or cultures with an interdependent self-construal or less independent self-construal. The proposal to include the fear of offending others in the DSM-V criteria of social phobia is supported by the present findings.

## Introduction

Taijin Kyofusho was introduced as a Japanese- or Korean-culture-bound form of social anxiety in the fourth edition of the Diagnostic and Statistical Manual of Mental Disorders (DSM-IV; APA, 1996, 2000) and as anthropophobia under social phobia in the tenth version of the International Classification of Mental and Behavioral Disorders [ICD-10; World Health Organization (WHO, 1992)]. DSM-IV describes the essential feature of TKS as persistent and excessive fears of giving offense to others in social situations by their physical characteristics, such as blushing, gaze, or one’s body odor. In TKS the social fear is focused on doing something that is embarrassing for (an)other(s) instead of doing something that is embarrassing for oneself, which is the case in DSM-IV social phobia (also called Social Anxiety Disorder). In ICD-10 this offensive variant of TKS is not described, but the fear of social contact (especially friends), extreme self-consciousness (concern about physical appearance, body odor, bushing), and the fear of contracting diseases. Further descriptions of TKS symptoms are that TKS is experienced when the individuals have face-to-face contact with other people with common symptoms of (a) fears of offending others by blushing, emitting offensive odors, and staring inappropriately; (b) fears of offending others by presenting an improper facial expression, a blemish, or physical defect; (c) strongly convinced of offending others; and (d) being obsessed with feelings of shame (Kirmayer, 1991; Maeda and Nathan, 1999). Thus, except from the description of the ICD-10 criteria, the main feature of TKS^1^ is the fear to offend others. Several TKS cases with offensive social fears were originally reported exclusively in Japan (Kirmayer, 1991) until some cases also began to be described in Korea (Kasahara, 1988; Lee and Oh, 1999). Given the rarity of cases reported from countries other than Japan and Korea, it had been taken for granted that TKS is a unique culture-bound syndrome.

Recently, however, it has been suggested that TKS symptoms can be found outside Japan and Korea as well. TKS symptoms were for example found in the United States of America (Clarvit et al., 1996; Choy et al., 2008; Kim et al., 2008). They thus might not be as culturally unique as previously assumed.

The way people construe themselves, often described as independent versus interdependent, might play a role in TKS symptoms, and therefore explain why such symptoms are found in cultures other than Japan and Korea. Interdependent self-construal emphasizes the relatedness of self to a collective, and the identification of self in terms of social roles and relationships. Independent self-construal emphasizes individual autonomy, and defines the self as a bounded and distinctive locus of awareness and action, separate from the collective (Markus and Kitayama, 1991). Several studies found a positive association between an interdependent self-construal and TKS, and a negative association between an independent self-construal and TKS (Dinnel et al., 2002; Essau et al., 2011; Norasakkunkit et al., 2012). Variations in self-construal are found across and within cultures, and even within individuals (Hong et al., 2001). Moreover, people can access multiple cultural scripts, primed by different contextual cues (Hong and Chiu, 2001; Ryder et al., 2011). Therefore, the interpersonal fear to embarrass others with one’s own behavior (TKS), associated with an interdependent self-construal, might occur in all individuals around the world construing themselves as (also) interdependent in certain context(s) or domains.

Apart from the description of several cases with TKS in cultures other than Japan, little is known about TKS in other countries. Even in so-called collectivistic countries, in which TKS symptoms might be expected based on the correlation between an interdependent self-construal (related to collectivistic cultures) and TKS (Triandis, 1989; Markus and Kitayama, 1991; Kim et al., 1994; Triandis et al., 1995), TKS is understudied. The present study investigates TKS symptoms in Indonesia, a so-called collectivistic country (Hofstede, 2001) with a population almost twice as big as Japan and therefore – perhaps – with twice as much cases with TKS. Hofstede’s (1980) ranking of individualism across cultures, Indonesia was ranked 47th out of the 53 countries and regions assessed and Indonesia was the least individualistic country of nine diverse nations assessed by Triandis et al. (1986). Also the Indonesian culture is a high power distance culture where the emphasis is on obedience, conformity, authority, supervision, social hierarchy, and inequality. The importance of group harmony and living together in harmony is emphasized in social relations (Hofstede, 2001). Interestingly, in Indonesia TKS-like symptoms have been described, such as a Javanese father of a patient with schizophrenia, who does not talk about his own feeling of shame but about the avoidance of the feeling of shame in others (Zaumseil and Lessmann, 2007). Also, Indonesian social emotions are described that include interpersonal feelings, such as is the case in TKS. For example, the Javanese term is in (meaning “shame,” “embarrassment,” “shyness,” and “respect,” which is very close to social anxiety; Geertz, 1959; Al Jallad, 2002) and the Balinese term lek (meaning “shame” and “stage fright”; Geertz, 1973; Keeler, 1983) encompasses both negative feelings of social anxiety and exposure as well as positive associations with proper social performance and cultivation of respect for authority, and the desire to avoid negative confrontation. Further, the Javanese adhere to an implicit social rule that face-to-face contact should be harmonious and polite (Keeler, 1987). Showing proper respect to others, keeping opinions to oneself, and being indirect in actions and words are highly valued in the culture (Mulder, 1992). Conversely, failure to maintain harmonious relationships often lead to feelings of shame for both parties (i.e., malu), indicating the interpersonal structure of social anxious feelings. Even more, the Indonesian term *malu* reflects both recognition of status inferiority and personal norm violations in social context, whereas shame in Western societies tends to emphasize the personal norm violations and resulting deficiencies in self-esteem (Fessler, 2004; Budden, 2009).

**Figure 1:**
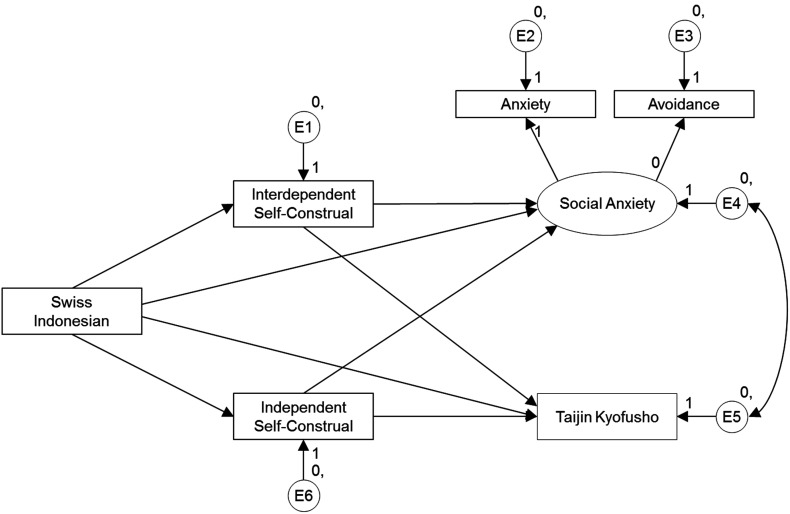
**Path model examining the associations between cultural background (Swiss/Indonesian), self-construal, social anxiety, and taijin kyofusho**.

Within this context, we hypothesize that TKS might be relevant in Indonesia as well and compare TKS and social anxiety symptoms, as well as their clinical relevance, in Indonesia with the same symptoms and their clinical relevance in Switzerland. The Swiss culture should be representative for a Western, rather individualistic culture (Hofstede, 2001), with extremely high rates of “Western” Social Phobia (Wacker et al., 1992). In a cultural value survey of Schwartz (1999) the Swiss sample attributed the most importance to intellectual autonomy values (curiosity, broadmindedness, creativity) of all the samples studied and attributed relatively high importance to affective autonomy and to egalitarianism values, but low importance to hierarchy values. In that survey the values of the Swiss sample showed to be opposite to those of the Indonesian sample, making these two samples interesting to compare. The clinical relevance of TKS, controlled for general social anxiety, is investigated by looking at the association of these symptoms with DSM-IV social phobia and with the wish for professional help for dealing with social fears. The study also examines how independent and interdependent self-construals are associated with TKS and/or if self-construal mediates the relationship between cultural background (Swiss versus Indonesian) and TKS (see Figure 1).

We expect higher scores for TKS in Indonesia compared to Switzerland. Also we expect higher social anxiety symptoms based on findings of higher degree of social anxiety symptoms in Asian samples compared to European and American samples (e.g., Dinnel et al., 2002; Lee et al., 2006; Hong and Woody, 2007). Furthermore, we hypothesize that in Indonesia TKS is more clinically relevant than in Switzerland. Based on the findings of Dinnel et al. (2002) we hypothesize that interdependent self-construal is positively associated and that independent self-construal is negatively associated with social anxiety. We expect that an Indonesian cultural background is associated with a more interdependent self-construal and a less independent self-construal, and that a Swiss cultural background is positively associated with independent self-construal and negatively associated with interdependent self-construal. Furthermore we hypothesize that self-construal mediates the association between people’s cultural background and TKS and social anxiety.

## Methods and Materials

### Sample

The present sample (*N* = 660) comprised two study groups of university students, one from Indonesia and one from Switzerland. Participants from the Indonesian study group (*N* = 311) were undergraduate psychology students from the Universitas Gadjah Mada (Jogjakarta, Indonesia). Participants from the Swiss study group (*N* = 349) were undergraduate psychology students from the University of Basel (Basel, Switzerland). In Table 1, participants’ age and gender ratios are presented. The Indonesian sample was significant younger than the Swiss sample. The unequal sex distribution (more than 75% female participants) was equal in both samples.

**Table 1:** **Differences on age, sex, and social anxiety between the Indonesian and Swiss study groups**.

	Indonesian sample	Swiss sample	Test		
	*n* = 311	*n* = 349	χ^2^	*t*	*p*
Sex (female, %)	76	78	0.242		n.s.
Age [*M* (SD)]	20.8 (3.2)	22.5 (5.3)	4.585		<0.001
Self-construal [*M* (SD)]					
Interdependent	3.89 (0.36)	3.22 (0.42)		22.71	<0.001
Independent	3.63 (0.41)	3.63 (0.39)		−0.150	n.s.
Social anxiety [*M* (SD)]	41.82 (19.07)	30.15 (17.14)		8.261	<0.001
Anxiety	21.26 (10.39)	14.29 (9.29)		9.082	<0.001
Avoidance	20.54 (9.72)	15.85 (9.59)		6.216	<0.001
Taijin kyofusho [*M* (SD)]	93.49 (29.97)	63.02 (26.57)		13.845	<0.001
Self-report DSM-IV social phobia (%)	15.8	6.2	13.911		<0.001
Wish for professional help for social anxiety (%)	21.4	14.5	5.260		0.022

**M*, mean; SD, standard deviation; *p*, *p*-value*.

### Questionnaire

We used a paper and pencil questionnaire battery consisting of self-report measures for self-construal, social anxiety, taijin kyofusho, DSM-IV social phobia, and the wish for professional help for social anxiety symptoms. For the translation of the Indonesian version of the questionnaires, we applied a translation-back translation procedure (Werner and Campbell, 1970). Two independent translators for each country, fluent in both Bahasa Indonesian and English, translated the complete questionnaire from English to Bahasa Indonesian. The third author (PN), fluent in Bahasa Indonesian, resolved inconsistencies in translation through deliberation with both translators. The Indonesian version of the questionnaire was then back-translated into English by another independent translator, who was fluent in both Bahasa Indonesian and English. As before, PN evaluated the translation by comparing the original and the back-translated version. No striking differences were detected between the two versions. For the Swiss version we used German translated and validated versions of the questionnaires if they were available. To obtain German versions of the remaining questionnaires (instructions, TKS, and clinical relevance), we used a similar translation-back translation procedure as with the Indonesian version.

We invited students of the two universities to fill out the questionnaires prior to a psychology lecture. They completed the questionnaire in the lecture room with a pencil they could keep as a gift. Completion of the questionnaires took about 15 min.

#### Self-construal

The Singelis Self-Construal Scale (Singelis, 1994) was used to measure self-construal. It consists of two 12-item sub-scales, assessing interdependent and independent self-construal. An example from the independent self-construal scale is: “I enjoy being unique and different from others in many respects” and from the interdependent self-construal is: “I will sacrifice my self-interest for the benefit of the group I am in.” The participants responded on a five-point Likert scale from “fully disagree” to “fully agree.” The mean score of the 12 items of each sub-scale, resulting in a score range from 1 to 5, was used. This widely used scale allows for comparison with other studies of cross-cultural self-construal. Multiple studies have shown the sub-scales to have either acceptable internal consistency (Singelis, 1994; Singelis and Sharkey, 1995; Sato and McCann, 1997; Norasakkunkit and Kalick, 2002), or low reliability estimates (Okazaki, 2000; Levine et al., 2003). In the present study, the Cronbach alpha coefficient for the independent self-construal sub-scale was 0.51 for the Indonesian sample and 0.55 for the Swiss sample. For interdependent self-construal sub-scale the Cronbach alpha coefficient for the Indonesian sample was 0.53 and for the Swiss sample was 0.62. These Cronbach alpha coefficients indicate moderate internal consistency.

*Social anxiety* was measured with the self-report version of the Liebowitz Social Anxiety Scale (LSAS: Liebowitz, 1987; Baker et al., 2002). Participants indicated on a 0 to 3 categorical sub-scale how much they fear and/or avoid 24 social situations, 13 relating to performance anxiety, and 11 concerning social interaction situations. The LSAS (where the clinician fills out the answers for the patient) and the LSAS-SR (a self-report version of the LSAS) have been found to have good psychometric properties (Heimberg et al., 1999; Fresco et al., 2001). In the present study, Cronbach’s alphas for the LSAS were 0.94 in Indonesia and 0.93 in Switzerland.

*Taijin kyofusho* was measured by the 31-item TKS (Kleinknecht et al., 1997). The items reflect symptoms that were found most highly discriminated TKS patients in Japan from non-patients and the items are consistent with descriptions of TKS’s definitional symptoms (Takahashi, 1989; Kirmayer, 1991; Nakamura and Shioji, 1997). The TKS reflects the respondents’ concerns that they would do something to offend or embarrass others. Responses are made on a seven-point Likert-type rating scale, ranging from totally false (1) to exactly true (7). For the analyses the sum score of these items (ranging from 31 to 217) was used. An example item is “I am afraid that eye-to-eye contact with other people will offend them.” The English and Japanese version of the taijin kyofusho have been demonstrated to possess sound internal consistency with Cronbach’s alphas of 0.92 and 0.93 for Japanese and American participants, respectively. In the present study, Cronbach’s alphas were 0.95 in Indonesia and 0.94 in Switzerland.

*Clinical relevance* was investigated with self-report DSM-IV social phobia and the wish for professional help for social fears. Self-report DSM-IV social phobia was measured with a question for each of the DSM-IV criteria of social phobia. The participants could answer “yes” or “no.” If all criteria of DSM-IV social phobia were answered with “yes,” the participant fulfilled “DSM-IV social phobia.” Wish for professional help for social anxiety symptoms was measured with one question: “Did you ever wish professional help (e.g., medication, counseling, psychotherapy, herbs) for social fears?” The participant could choose the answers “yes” and “no.”

### Analyses

Differences between study groups were tested with χ^2^- and *t*-tests performed on SPSS Version 20 (IBM coop). The association between TKS and self-report DSM-IV social phobia, controlled for social anxiety, and the association between TKS and the wish for professional help for social fears, controlled for social anxiety and self-report DSM-IV social phobia, were tested with binary logistic regression analyses performed by SPSS Version 20 (IBM coop) with all independent variables entered in the model.

The mediation model was analyzed with AMOS Version 5.0 (Arbuckle, 2003). Maximum likelihood method was used for estimation. Model fit was tested using GFI, CFI, and RMSEA. GFI and CFI values higher than 0.95 indicate that the model explains the variance of the data well (Kline, 1998). RMSEA provides a fit index unaffected by the size of the model by considering degree of freedom; an RMSEA of 0.08 or lower is conventionally considered to be an acceptable value. Not significant paths were removed from the model until all paths were significant. For all analyses the significance level was *p* < 0.05.

## Results

### Cultural and social anxiety differences

The Indonesian sample scored significant higher on interdependent but not on independent self-construal compared to the Swiss sample (see Table 1). The Indonesian sample reported more social anxiety symptoms, and more TKS symptoms than the Swiss sample (see Table 1). Also in the Indonesian sample more individuals fulfilled the self-report DSM-IV social phobia criteria compared to the Swiss sample. The wish for professional help to deal with social fears was more frequent in the Indonesian sample than in the Swiss sample (see Table 1).

### DSM-IV social phobia and wish for professional help

Fulfillment of the self-report criteria of DSM-IV social phobia was in the Indonesian sample predicted by higher scores for social anxiety and for TKS, but in the Swiss sample only with higher scores for social anxiety (see regression coefficients Table 2)^2^. Wish for professional support for social anxiety symptoms was in the Indonesian sample predicted by fulfillment of self-report DSM-IV social phobia and with higher scores for TKS. In the Swiss sample, the wish for professional support for social anxiety symptoms was only predicted self-report DSM-IV social phobia (see Table 2; see text footnote 1).

**Table 2:** **Logistic regression for associations with the dependent variables “self-report DSM-IV social phobia” and “wish for professional help for social anxiety symptoms” in the Indonesian (*N* = 311) and Swiss sample (*N* = 344)**.

Associations	Indonesian sample		Swiss sample	
	β	95% CI	β	95% CI
**DSM-IV SOCIAL PHOBIA**				
Social anxiety	**1.034**	**1.011–1.057**	**1.053**	**1.027–1.080**
Taijin kyofusho	**1.019**	**1.005–1.034**	1.012	0.948–1.027
**WISH FOR PROFESSIONAL HELP**				
DSM-IV social phobia	**4.200**	**1.746–10.106**	**12.081**	**4.214–34.633**
Social anxiety	0.984	0.963–1.006	1.003	0.982–1.025
Taijin kyofusho	**1.021**	**1.008–1.035**	1.009	0.996–1.021

*β, Expected beta; 95% CI, 95% confidence interval; DSM-IV social phobia, self-report DSM-IV social phobia; wish for professional help, wish for professional help for social anxiety symptoms; social anxiety, Liebowitz Social Anxiety Scale score; taijin kyofusho, Taijin Kyofusho Scale score, significant β’s are printed bold type*.

### Associations and mediation between cultural background, self-construal, social anxiety, and taijin kyofusho

**Figure 2:**
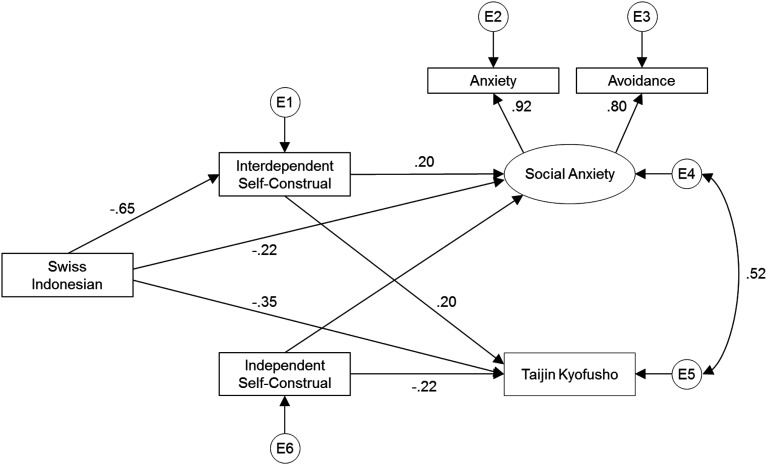
**Standardized estimates of the path model with the associations between cultural background (Swiss/Indonesian), self-construal, social anxiety, and taijin kyofusho**.

Our model as depicted in Figure 1 revealed a zero, non-significant association between cultural background and an independent self-construal as could be expected based on the equal scores of the study groups on “independent self-construal” (see Table 1). Therefore we rerun the model without that association. This model (see Figure 2) showed excellent fit, with a χ^2^ = 6.679 (*p* = 0.246), CFI of 0.999, and RMSEA of 0.023. All associations in the model were significant (all *p*s < 0.001). As can be seen from the model in Figure 2, Social Anxiety and TKS were strongly correlated (*r* = 0.52). Indonesian background predicted higher scores for social anxiety and for TKS (respect. *r* = −22, *r* = −0.35). The effect of cultural background on social anxiety and TKS was partially mediated by an interdependent self-construal. The indirect effect of cultural background on social anxiety was −0.103 (standardized −0.128). The indirect effect of cultural background on TKS was −8.199 (standardized 0.129). An interdependent self-construal predicted higher score on social anxiety and TKS (*r* = 0.20 and *r* = 20), and an independent self-construal predicted lower scores on social anxiety and TKS (*r* = −0.36 and *r* = −0.22).

## Discussion

In line with our hypothesis the present study indicates that TKS might not only be a Japanese culture-bound disorder, but also be present in other cultures or individuals, that are collectivistic. The TKS values of the present Indonesian sample were higher compared to the Swiss sample and had similar values as observed in Japanese samples (Dinnel et al., 2002; Essau et al., 2011). In Indonesia TKS showed to be clinically relevant, as it was associated with self-report DSM-IV social phobia (even when controlling for social anxiety) and with the wish for professional help to deal with social fears. On the contrary in Switzerland TKS was not associated with self-report DSM-IV social phobia and not with the wish for professional help to deal with social fears, indicating that TKS is probably not (or at least less) clinically relevant in Switzerland. In line with other studies (Dinnel et al., 2002; Essau et al., 2011; Norasakkunkit et al., 2012) we found that interdependent self-construal was positively associated with TKS and social anxiety, and that an independent self-construal was negatively associated with TKS and social anxiety. Interdependent self-construal mediated the association between cultural background and TKS and social anxiety – i.e., a more interdependent self-construal, associated with an Indonesian cultural background, was associated with TKS and social anxiety. Thus, TKS might not be a Japanese culture-bound syndrome, but an “interdependent-self-construal-bound-syndrome.” Those individuals, who construe themselves as interdependent with others, have a greater chance to experience social fears that rely on interdependence, such as fearing to embarrass others with one’s own behavior. According to the present model, it can be assumed that in Indonesia and in Switzerland TKS symptoms are more often present in individuals with a more interdependent and a less independent self-construal compared to an individual with a more independent and less interdependent self-construal. Also the model suggests that in Indonesia this is more often the case in individuals, and thus more cases with TKS can be found than in Switzerland. However, in Switzerland some cases with TKS might also be found.

We found a high rate of self-report social phobia in Indonesia (15.8%), which is in contrast with epidemiological studies in Asia that found very low prevalence rates for DSM-IV social phobia (0.5–1.2%; Hwu et al., 1989; Lee et al., 1990; Tsuchiya et al., 2009). There are several explanations for the present high rate of social phobia in Indonesia. First, this rate of DSM-IV social phobia is in line with studies examining the frequency of social anxiety symptoms that have almost always reported higher levels of these symptoms among participants from Asia compared to participants from Western countries (e.g., Kleinknecht et al., 1997; Dinnel et al., 2002; Heinrichs et al., 2006; Lee et al., 2006; Hong and Woody, 2007). Some research groups are trying to explain the existing contrast between results of questionnaire studies with high self-report social anxiety symptoms and results of epidemiological studies with low prevalence rates of clinically relevant DSM-IV social phobia (Heinrichs et al., 2006; Essau et al., 2011). However, to our knowledge, nobody investigated both (DSM-IV social phobia and self-report social anxiety symptoms questionnaires) in the same community sample. Our study also does not compare DSM-IV social phobia rated by an external rater as it is done in epidemiological studies. But it comes close to this by investigating self-report DSM-IV social phobia. The validity of this kind of measurement might not be severely compromised, as the estimate of present social phobia in Switzerland (6.2%) is very close to the prevalence rates of epidemiological studies in the West (Ruscio et al., 2008). Thus, an explanation for our finding would be that there is a contrast in self-report versus other-report methods in Indonesia instead of a real contrast between DSM-IV social phobia and social anxiety symptoms. This explanation would be culture-specific, because in Switzerland self-report SAD was close to SAD rates of epidemiological studies. It is conceivable that in Indonesia, cultural variables such hierarchy, harmony, and interdependence play a role in different results for self-, other-, or interview-report. Another explanation for the high rates of self-report SAD, which adds to the previous one, is that the participants of the present study were primed by the questionnaires of social anxiety symptoms when they filled out the DSM-IV social phobia criteria. As they were asked about many social situations, social fears, including TKS symptoms, they might have been more conscious about their fears at the moment they filled out the DSM-IV social phobia criteria, as if they would be in a standardized clinical interview, which usually has only six items with social situations that are feared and/or avoided. A third explanation is that in Indonesia an epidemiological study would find as high DSM-IV social phobia prevalence rates as we did (in contrast with other Asian epidemiological studies). Unfortunately, no such study has been published so far to compare our results with.

In Indonesia both social anxiety and TKS symptoms were higher compared to the Swiss sample. This is in line with studies that found higher social anxiety symptoms in Asian compared to Western cultures (e.g., Kleinknecht et al., 1997; Dinnel et al., 2002; Heinrichs et al., 2006; Lee et al., 2006; Hong and Woody, 2007) and with recent studies reporting strong correlations between scores on the Social Phobia Scale (SPS) and Social Interaction and Anxiety Scale (SIAS) and scores on the measures of TKS (Choy et al., 2008; Essau et al., 2011) or fear of causing discomfort to others (Rector et al., 2006) in patients with social phobia. These high correlations suggest that social anxiety and TKS might represent one latent construct. In both TKS and social anxiety (as it is known in the West) the central fear is being evaluated negatively. In TKS this fear of being evaluated negatively is based on the fear of doing something that could offend (an)other person(s), and in social anxiety, as it is known in the West, the fear is based on doing something that is embarrassing for oneself (without offending others). An argument for this hypothesis is that Essau et al. (2011) found a strong correlation (*r* = 0.74) between TKS and the Fear of Negative Evaluation. They and others also suggest that TKS may be a variant of social interaction anxiety or that these different forms of social anxiety represent a single higher order entity (Lee and Oh, 1999; Nakamura et al., 2002; Nagata et al., 2006). If indeed TKS is found across many cultures (probably mediated by cultural variables such as interdependent self-construal), and is another form of social anxiety, offensive social fears should be included in the DSM-V criteria for social anxiety disorder. Indeed, for DSM-V, it has been proposed to change Criterion B into “The individual fears that he or she will act in a way or show anxiety symptoms that will be negatively evaluated (e.g., be humiliated, embarrassed, or rejected) or will offend others” (www.dsm5.org).

The present study has some limitations. First, the results cannot be generalized to the general populations of Indonesia or Switzerland, as they are based on student, mainly female, samples. For example, it is possible that psychology students are less naive about the nature of psychology experiments and may produce a biased or unrepresentative sample even of the student population. Future studies must replicate these findings in general (less educated) populations and clinical samples. Second, our finding that independent self-construal was not more pronounced in the Swiss than in the Indonesian sample was somewhat surprising, because this is contrary to what traditional conceptualizations of culture might suggest. However, nowadays, in other so-called collectivistic cultures, such as Japan and Korea, independent self-construals similar to the ones in the United States of America have been found (Yang et al., 2006; Vriends and Halim, 2009). In addition, some other studies found as well that Japanese and North-American samples did not differ on the interdependent self-construal scale of Singelis (Dinnel et al., 2002; Norasakkunkit and Kalick, 2002; Norasakkunkit et al., 2012). Thus, self-construal might currently change with increased exposure to each other’s cultural values as a result of technology and increased overseas travel. Also, attitudinal measures of culture are vulnerable to reporting biases (see for an overview (Kitayama, 2002), such as reference group effects, challenging us to investigate cultural attitudes properly in cultural settings characterized by rapid change. Third, the DSM-IV social phobia was based on self-report. Usually, a clinician diagnoses DSM-IV social phobia. It would be interesting (and of course time-consuming) to compare our findings with findings based on the fulfillment of these criteria, judged by a clinician. Fourth, reliability rates of the self-construal scales were only moderate. This precludes definite conclusions regarding the mediation effect of interdependent self-construal and regarding associations of self-construal with TKS. Also the TKS questionnaire that we used was developed in Japan and has not been formally validated for use in Indonesia. The present results indicate that might be valuable to do this.

What are the implications of our results despite these limitations? To date, research on social fears or social anxiety in Indonesia has been largely neglected. If our findings are replicated in the general population from Indonesia, clinicians in Indonesia should become more aware of social anxiety and TKS symptoms. Despite Indonesia being the fourth largest nation in the world with 237 million inhabitants, a literature search for the keywords “social anxiety disorder” or “social phobia” and “Indonesia” yielded only four publications in PubMed and zero publications in Psychinfo^3^. Even when estimating the number of Indonesian people with social phobia based on a very low estimated prevalence rate of 2%, we would need to assume that five million people are affected by the disorder. As suggested by the present findings, social anxiety (including TKS symptoms) may be a clinically relevant problem in Indonesia, although it has been neglected in a way similar to the way it has been neglected in Western societies until the 1980s (Liebowitz et al., 1985).

The discrepancy between the present high rate of self-report SAD and TKS symptoms in Indonesia and its neglect in research and clinical settings might also have Indonesian-specific reasons; in Indonesia, there is only one psychiatrist per 10 million inhabitants (WHO, 2011)^4^. Thus other majoring problems, such as psychosis, post-traumatic stress disorder, obsessive compulsive disorders, and substance-dependence might have their logical priority. However, social anxiety symptoms usually precede other psychiatric disorders (Wittchen and Fehm, 2003) and might be seen as the first sign of a “psychiatric career.” Therefore, recognizing and treating social anxiety symptoms may prevent people from having functional and personal impairments, which are found to be associated with untreated social anxiety, but might also have more general mental health benefits in a collectivistic society such as Indonesia.

The contrast between our findings and the neglect of social anxiety research in Indonesia might also reflect the well-known medical anthropology’s distinction between illness and disease, in which illness is the patient’s perception, experience, and communication of symptoms, while disease is the clinician’s reformulation of the problem in terms of psychiatric models (Kleinman, 1987). Future research might try to map carefully the culture-bound distress of social anxiety and its consequences.

Future research might also explore the way of help-seeking for psychological disorders the cultural contexts. Cultures have culture-specific associations with professional help-seeking (Kurihara et al., 2000; Setiawan, 2006). For example in Indonesia people usually visit traditional healers (e.g., *dukun*) and use spiritual powders and herbal medicine (e.g., *jamu, jin*), even simultaneously to modern medical care (Subandi, 2009) for their complaints. Future research might investigate if Indonesian respondents include such indigenous healers in their lay notions of professional help-seeking. Besides exploring variation in help-seeking, it would also be relevant to compare self-report help-seeking rates with objective help-seeking rates (e.g., percentage of patients in clinics having social anxiety or TKS symptoms). Related to this, clinical relevance of TKS and social anxiety symptoms might also be investigated with other measurements such as number and cost of medical usage (e.g., number of visits to general practitioner or traditional healer, medication use) or personal and functional impairment due to symptoms.

Also, it needs to be investigated if development and maintenance models of social phobia need to be extended to make therapies effective for both fears, the fear to embarrass oneself and the fear to embarrass (or offend) others. For example, Vriends et al. (under review) found that an interdependent self-construal is associated with increased other-focused attention instead of increased self-focused attention, as proposed in existing maintenance models of social phobia (Clark and Wells, 1995). On the other hand, Kim et al. (2008) found that TKS symptoms in Australian patients with DSM-IV social phobia decreased after treatment for social phobia. Perhaps behavioral therapy interventions such as exposure therapy have similar effects on both types of fears. Namely, they may decrease avoidance behavior through which corrective experiences can be made. Cognitive therapy might focus on different, fear-specific cognitive schemata. Similarly, the focus of attention therapy (more attention toward oneself versus more attention toward the task or surrounding) may perhaps need to vary, depending on the type of fear.

Further, the possible complexity of the relationship between an interdependent context and TKS symptoms needs to be investigated. It is likely that there are significant differences within interdependent context – not all people scoring high on collectivism are alike. For example, interdependent contexts seem to differ in their emphasis on harmony, and TKS concerns may be fueled in part by valuing harmony (Hui and Triandis, 1985; Schwartz, 1999). Thus, future research might question what the more proximal cultural factors are that promote this type of social concerns.

In summary, TKS symptoms seem to be common and clinically relevant in Indonesia. Our data suggest that TKS symptoms are likely related to interdependent self-construal, which is present in Indonesia. However, TKS might be present in cultures and in individuals with an interdependent self-construal all around the world. Our results support the proposed change in DSM-V to include “offend others” in the criteria of social phobia. Furthermore, they stress the need for further research into social anxiety and TKS in other collectivistic cultures, and particularly in Indonesia.

## Conflict of Interest Statement

The authors declare that the research was conducted in the absence of any commercial or financial relationships that could be construed as a potential conflict of interest.

## Footnotes

^1^Due to focus of this paper we refer in this manuscript to TKS, as defined in DSM-IV, which is probably close to the conviction subtype of TKS, also called the offensive subtype of TKS.

^2^This analysis was first controlled for age. Because age did not have an association with DSM-IV social phobia, we present results for the logistic regression analysis without age.

^3^The same terms find 11 items in Psychinfo and 141 items in PubMed for Switzerland (7.6 million inhabitants).

^4^In Switzerland there are 41 psychiatrist per 100,000 inhabitants, thus 4100 per 10,000,000 inhabitants.

## References

[B1] Al Jallad N. T. (2002). Same in English, Arabic and Javanese: A Comparative Lexical Study. Ph.D. thesis, Dissertation, University of Delaware, Newark, Delaware

[B2] APA. (1996). Diagnostic and Statistical Manual of Mental Disorders, 4th Edn Washington, DC: American Psychiatric Association

[B3] APA. (2000). Diagnostic and Statistical Manual of Mental Disorders, 4th Edn, Text Revision. Washington, DC: American Psychiatric Association

[B4] Arbuckle J. L. (2003). AMOS 5.0 [Computer Software]. Chicago: SPSS

[B5] Baker S. L. Heinrichs N. Kim H. J. Hofmann S. G. (2002). The liebowitz social anxiety scale as a self-report instrument: a preliminary psychometric analysis. Behav. Res. Ther. 40, 701–715 10.1016/S0005-7967(01)00060-2 12051488

[B6] Budden A. (2009). The role of shame in posttraumatic stress disorder: a proposal for a socio-emotional model for DSM-V. Soc. Sci. Med. 69, 1032–1039 10.1016/j.socscimed.2009.07.032 19695754

[B7] Choy Y. Schneier F. R. Heimberg R. G. Oh K. S. Liebowitz M. R. (2008). Features of the offensive subtype of Taijin-Kyofu-Sho in US and Korean patients with DSM-IV social anxiety disorder. Depress. Anxiety 25, 230–240 10.1002/da.20295 17340609

[B8] Clark D. M. Wells A. (1995). A Cognitive Model of Social Phobia. New York: The Guilford Press

[B9] Clarvit S. R. Schneier F. R. Liebowitz M. R. (1996). The offensive subtype of Taijin-Kyofu-Sho in New York City: the phenomenology and treatment of a social anxiety disorder. J. Clin. Psychiatry 57, 523–527 10.4088/JCP.v57n1104 8968301

[B10] Dinnel D. L. Kleinknecht R. A. Tanaka-Matsumi J. (2002). A cross-cultural comparison of social phobia symptoms. J. Psychopathol. Behav. Assess. 24, 75–84 10.1023/A:1015316223631

[B11] Essau C. A. Sasagawa S. Chen J. Sakano Y. (2011). Taijin kyofusho and social phobia symptoms in young adults in England and in Japan. J. Cross Cult. Psychol. 43, 219–232 10.1177/0022022110386372

[B12] Fessler D. M. T. (2004). Shame in two cultures: implications for evolutionary approaches. J. Cogn. Cult. 4, 207–262 10.1163/1568537041725097

[B13] Fresco D. M. Coles M. E. Heimberg R. G. Liebowitz M. R. Hami S. Stein M. B. (2001). The Liebowitz Social Anxiety Scale: a comparison of the psychometric properties of self-report and clinician-administered formats. Psychol. Med. 31, 1025–1035 10.1017/S0033291701004056 11513370

[B14] Geertz C. (1973). The Interpretation of Cultures. New York: Basic Books

[B15] Geertz H. (1959). The vocabulary of emotion: a study of Javanese socialization processes. Psychiatry 22, 225–237 13827079 10.1080/00332747.1959.11023175

[B16] Heimberg R. G. Horner K. J. Juster H. R. Safren S. A. Brown E. J. Schneier F. R. (1999). Psychometric properties of the Liebowitz Social Anxiety Scale. Psychol. Med. 29, 199–212 10.1017/S0033291798007879 10077308

[B17] Heinrichs N. Rapee R. M. Alden L. A. Bogels S. Hofmann S. G. Oh K. J. (2006). Cultural differences in perceived social norms and social anxiety. Behav. Res. Ther. 44, 1187–1197 10.1016/j.brat.2005.09.006 16325145

[B18] Hofstede G. (1980). Culture’s Consequences: International Differences in Work-Related Values. Beverly Hills: Sage Publications

[B19] Hofstede G. (2001). Culture’s Consequences: Comparing Values, Behaviors, Institutions and Organizations Across Nations. Thousand Oaks: Sage Publications

[B20] Hong J. J. Woody S. R. (2007). Cultural mediators of self-reported social anxiety. Behav. Res. Ther. 45, 1779–1789 10.1016/j.brat.2007.01.011 17350589

[B21] Hong Y. Y. Chiu C. Y. (2001). Toward a paradigm shift: from cross-cultural differences in social cognition to social-cognitive mediation of cultural differences. Soc. Cogn. 19, 181–196 10.1521/soco.19.3.181.21471

[B22] Hong Y. Y. Ip G. Chiu C. Y. Morris M. W. Menon T. (2001). Cultural identity and dynamic construction of the self: collective duties and individual rights in Chinese and American cultures. Soc. Cogn. 19, 251–268 10.1521/soco.19.3.181.21471

[B23] Hui C. H. Triandis H. C. (1985). Measurement in cross-cultural psychology – a review and comparison of strategies. J. Cross Cult. Psychol. 16, 131–152 10.1177/0022002185016002001

[B24] Hwu H. G. Yeh E. K. Chang L. Y. (1989). Prevalence of psychiatric-disorders in taiwan defined by the chinese diagnostic interview schedule. Acta Psychiatr. Scand. 79, 136–147 10.1111/j.1600-0447.1989.tb08581.x 2923007

[B25] Kasahara Y. (1988). Social phobia in Japan. Transcult. Psychiatry 25, 145–150 10.1177/136346158802500213

[B26] Keeler W. (1983). Shame and stage fright in java. Ethos 11, 152–165 10.1525/eth.1983.11.3.02a00040

[B27] Keeler W. (1987). Shadow world of the Javanese. Nat. Hist. 96, 68

[B28] Kim J. Rapee R. M. Gaston J. E. (2008). Symptoms of offensive type Taijin-Kyofusho among Australian social phobics. Depress. Anxiety 25, 601–608 10.1002/da.20345 17607747

[B29] Kim U. Triandis H. C. Kâgitçibasi Ç. Choi S. Yoon G. (1994). Individualism and Collectivism: Theory, Method, and Applications, Vol. 18 Thousand Oaks, CA: Sage Publications, Inc

[B30] Kirmayer L. J. (1991). The place of culture in psychiatric nosology – Taijin-Kyofusho and Dsm-III-R. J. Nerv. Ment. Dis. 179, 19–28 10.1097/00005053-199101000-00005 1985144

[B31] Kitayama S. (2002). Culture and basic psychological processes – toward a system view of culture: comment on Oyserman et al. (2002). Psychol. Bull. 128, 89–96 10.1037/0033-2909.128.1.89 11843550

[B32] Kleinknecht R. A. Dinnel D. L. Kleinknecht E. E. Hiruma N. Harada N. (1997). Cultural factors in social anxiety: a comparison of social phobia symptoms and Taijin kyofusho. J. Anxiety Disord. 11, 157–177 10.1016/S0887-6185(97)00004-2 9168340

[B33] Kleinman A. (1987). Anthropology and psychiatry – the role of culture in cross-cultural research on illness. Br. J. Psychiatry 151, 447–454 10.1192/bjp.151.4.447 3447661

[B34] Kline R. B. (1998). Principles and Practice of Structural Equation Modeling. New York: Guilford

[B35] Kurihara T. Kato M. Sakamoto S. Reverger R. Kitamura T. (2000). Public attitudes towards the mentally ill: a cross-cultural study between Bali and Tokyo. Psychiatry Clin. Neurosci. 54, 547–552 10.1046/j.1440-1819.2000.00751.x 11043804

[B36] Lee C. K. Kwak Y. S. Yamamoto J. Rhee H. Kim Y. S. Han J. H. (1990). Psychiatric epidemiology in Korea. Part II: Urban and rural differences. J. Nerv. Ment. Dise. 178, 247–252 10.1097/00005053-199004000-00005 2181056

[B37] Lee C. K. Oh K. S. (1999). Offensive type of social phobia: cross-cultural perspectives. Intern. Med. J. 6, 271–279

[B38] Lee M. R. Okazaki S. Yoo H. C. (2006). Frequency and intensity of social anxiety in Asian Americans and European Americans. Cultur. Divers. Ethnic Minor. Psychol. 12, 291–305 10.1037/1099-9809.12.2.291 16719578

[B39] Levine T. R. Bresnahan M. J. Lapinski M. K. Wittenbaum G. M. Morinagashearman S. Lee S. Y. (2003). Self-construal scales lack validity. Hum. Commun. Res. 29, 210–252 10.1111/j.1468-2958.2003.tb00840.x

[B40] Liebowitz M. R. (1987). Social phobia. Mod. Probl. Pharmacopsychiatry 22, 141–173 2885745 10.1159/000414022

[B41] Liebowitz M. R. Gorman J. M. Fyer A. J. Klein D. F. (1985). Social phobia. Review of a neglected anxiety disorder. Arch. Gen. Psychiatry 42, 729–736 10.1001/archpsyc.1985.01790300097013 2861796

[B42] Maeda F. Nathan J. H. (1999). Understanding taijin kyofusho through its treatment, Morita therapy. J. Psychosom. Res. 46, 525–530 10.1016/S0022-3999(98)00113-5 10454167

[B43] Markus H. R. Kitayama S. (1991). Culture and the self – implications for cognition, emotion, and motivation. Psychol. Rev. 98, 224–253 10.1037/0033-295X.98.2.224

[B44] Mulder N. (1992). Individual and Society in Java: A Cultural Analysis. Yogyakarta: Gadjah Mada University Press

[B45] Nagata T. van Vliet I. Yamada H. Kataoka K. Iketani T. Kiriike N. (2006). An open trial of paroxetine for the “offensive subtype” of taijin kyofusho and social anxiety disorder. Depress. Anxiety 23, 168–174 10.1002/da.20153 16456863

[B46] Nakamura K. Shioji R. (1997). Taijin kyoufushou to hikikomori [Taijin-kyofusho and withdrawal]. Rinshou Seishin Igaku 26, 1169–11176

[B47] Nakamura K. Kitanishi K. Miyake Y. Hashimoto K. Kubota M. (2002). The neurotic versus delusional subtype of taijin-kyofu-sho: their DSM diagnoses. Psychiatry Clin. Neurosci. 56, 595–601 10.1046/j.1440-1819.2002.01061.x 12485300

[B48] Norasakkunkit V. Kalick S. M. (2002). Culture, ethnicity, and emotional distress measures – the role of self-construal and self-enhancement. J. Cross Cult. Psychol. 33, 56–70 10.1177/0022022102033001004

[B49] Norasakkunkit V. Kitayama S. Uchida Y. (2012). Social anxiety and holistic cognition: self-focused social anxiety in the United States and other-focused social anxiety in Japan. J. Cross Cult. Psychol. 43, 742–757 10.1177/0022022111405658

[B50] Okazaki S. (2000). Asian American and white American differences on affective distress symptoms – do symptom reports differ across reporting methods? J. Cross Cult. Psychol. 31, 603–625 10.1177/0022022100031005004

[B51] Rector N. A. Kocovski N. L. Ryder A. G. (2006). Social anxiety and the fear of causing discomfort to others: rejoinder to Roth Ledley and Magee et al. J. Soc. Clin. Psychol. 25, 937–944 10.1521/jscp.2006.25.8.937

[B52] Ruscio A. M. Brown T. A. Chiu W. T. Sareen J. Stein M. B. Kessler R. C. (2008). Social fears and social phobia in the USA: results from the National Comorbidity Survey Replication. Psychol. Med. 38, 15–28 10.1017/S0033291707001699 17976249 PMC2262178

[B53] Ryder A. G. Ban L. M. Chentsova-Dutton Y. E. (2011). Towards a cultural–clinical psychology. Soc. Personal. Psychol. Compass 5, 960–975 10.1111/j.1751-9004.2011.00404.x

[B54] Sato T. McCann D. (1997). Vulnerability factors in depression: the facets of sociotropy and autonomy. J. Psychopathol. Behav. Assess. 19, 41–62 10.1007/BF02263228

[B55] Schwartz S. H. (1999). A theory of cultural values and some implications for work. Appl. Psychol. 48, 23–47 10.1111/j.1464-0597.1999.tb00047.x

[B56] Setiawan J. L. (2006). Willingness to seek counselling, and factors that facilitate and inhibit the seeking of counselling in Indonesian undergraduate students. Br. J. Guid. Counc. 34, 403–419 10.1080/03069880600769654

[B57] Singelis T. M. (1994). The measurement of independent and interdependent self-construals. Pers. Soc. Psychol. Bull. 20, 580–591 10.1177/0146167294205014

[B58] Singelis T. M. Sharkey W. F. (1995). Culture, self-construal, and embarrassability. J. Cross Cult. Psychol. 26, 622–644 10.1177/002202219502600607

[B59] Subandi M. A. (2009). Indegenous processes of recovery from psychosis in Java. Paper presented at the Workshop on Mental Health System Development for the Severe mental Illness in Asian Countries, Taipei Medical University, Taiwan

[B60] Takahashi T. (1989). Social phobia syndrome in Japan. Compr. Psychiatry 30, 45–52 10.1016/0010-440X(89)90117-X 2647401

[B61] Triandis H. C. Bontempo R. Betancourt H. Bond M. Leung K. Brenes A. (1986). The measurement of etic aspects of individualism and collectivism across cultures. Aust. J. Psychol. 38, 257–267 10.1080/00049538608259013

[B62] Triandis H. C. (1989). The self and social behavior in differing cultural context. Psychol. Rev. 96, 506–520 10.1037/0033-295X.96.3.506

[B63] Triandis H. C. Chan D. K. S. Bhawuk D. P. S. Iwao S. Sinha J. B. P. (1995). Multimethod probes of allocentrism and idiocentrism. Int. J. Psychol. 30, 461–480 10.1080/00207599508246580

[B64] Tsuchiya M. Kawakami N. Ono Y. Nakane Y. Nakamura Y. Tachimori H. (2009). Lifetime comorbidities between phobic disorders and major depression in Japan: results from the World Mental Health Japan 2002-2004 Survey. Depress. Anxiety 26, 949–955 10.1002/da.20508 19195005 PMC3641513

[B65] Vriends N. Halim M. (2009). Cultural differences in social anxiety disorder: how are they explained? Talk Presentation at the 39th Annual EABCT Congress, Dubrovnik

[B66] Wacker H. R. Müllejans R. Klein K. H. Battegay R. (1992). Identification of cases of anxiety disorders and affective disorders in the community according to ICD-10 and DSM-III-R using the Composite International Diagnostic Interview (CIDI). Int. J. Methods Psychiatr. Res. 2, 91–100

[B67] Werner O. Campbell D. (1970). “Translating, working through interpreters and the problem of decentering,” in Handbook of Cultural Anthropology, eds Naroll R. Cohen R. (New York: American Museum of National History), 398–419

[B68] WHO. (1992). International Statistical Classification of Diseases and Health Related Problems – 10th revision. Geneva: World Health Organization

[B69] WHO. (2011). Mental Health Atlas 2011. Geneva: World Health Organization

[B70] Wittchen H. U. Fehm L. (2003). Epidemiology and natural course of social fears and social phobia. Acta Psychiatr. Scand. 108, 4–18 10.1034/j.1600-0447.2003.00126.x 12950432

[B71] Yang R. P. J. Noels K. A. Saumure K. D. (2006). Multiple routes to cross-cultural adaptation for international students: mapping the paths between self-construals, English language confidence, and adjustment. Int. J. Intercult. Relat. 30, 487–506 10.1016/j.ijintrel.2005.11.010

[B72] Zaumseil M. Lessmann H. (2007). Dealing with schizophrenia in central java. Forum Gemeindepsychol. 12

